# Awareness of Direct-to-Consumer Genetic Tests and Use of Genetic Tests Among Puerto Rican Adults, 2009

**Published:** 2011-08-15

**Authors:** Ana Patricia Ortiz, Magdalena López, Libertad T. Flores, Marievelisse Soto-Salgado, Lila J. Finney Rutten, Ruby A. Serrano-Rodriguez, Bradford W. Hesse, Guillermo Tortolero-Luna

**Affiliations:** Department of Biostatistics and Epidemiology, Graduate School of Public Health, Medical Sciences Campus, University of Puerto Rico. Dr Ortiz is also affiliated with the University of Puerto Rico Comprehensive Cancer Center, San Juan, Puerto Rico; University of North Carolina at Chapel Hill, Chapel Hill, North Carolina; Alpert Medical School of Brown University, Providence, Rhode Island; University of Puerto Rico, San Juan, Puerto Rico; SAIC-Frederick, Inc, National Cancer Institute, Frederick, Maryland; Puerto Rico Department of Health, San Juan, Puerto Rico; National Cancer Institute, Bethesda, Maryland; University of Puerto Rico Comprehensive Cancer Center, San Juan, Puerto Rico

## Abstract

**Introduction:**

Genetic testing remains low among racial/ethnic minority populations in the United States. We aimed to determine the prevalence and correlates of awareness of direct-to-consumer (DTC) genetic tests and the prevalence of genetic test use in a population-based sample of adults in Puerto Rico.

**Methods:**

We analyzed data from adults aged 18 years or older who completed information on genetic test awareness (n = 611; 96% of study population) from the Health Information National Trends Survey conducted in Puerto Rico in 2009. Odds ratios with 95% confidence intervals were estimated by using logistic regression models to identify factors associated with awareness of DTC genetic tests.

**Results:**

The majority of respondents (56%) were aware of direct-to-consumer genetic tests, and approximately 4% had ever undergone any genetic test. Respondents who had never been married were less likely to be aware of DTC tests, as were current smokers. Respondents who ever sought cancer information were more likely to be aware of these tests.

**Conclusion:**

We provide the first published data on the awareness of DTC genetic tests and on use of genetic testing in Puerto Rico. Forty-four percent of our sample of Puerto Rican adults were unaware of direct-to-consumer genetic tests. Given the lack of clear benefits of DTC genetic tests to the general population, educational interventions should be developed to increase awareness and specific knowledge regarding the appropriate use of DTC genetic tests among people who are already aware of their existence.

## Introduction

With the completion of the Human Genome Project (1), genetic tests have increasingly become available to the population. Genetic testing is defined as "the analysis of human DNA, RNA, chromosomes, proteins, and certain metabolites in order to detect heritable disease-related genotypes, mutations, phenotypes, or karyotypes for clinical purposes" (2). Genetic tests that target specific diseases, such as cancer, are offered in health care settings to identify people with increased disease risk, for example, because of family history (3). The American Society of Clinical Oncology recognizes that identifying people at highest risk for cancer may improve prevention and early detection efforts and therefore supports access to genetic testing for all people with apparent heredity risk for cancer (4). In addition, the general public, patient groups, and people with a family history of cancer (5-7) appear to be strongly interested in genetic testing.

Despite increasing availability and public interest, studies on genetic testing are few, and most have focused on specific cancer types, as disease-specific tests have become more available in recent years. Previous research suggests that not all sociodemographic groups have benefited equally from genetic counseling and testing for cancer susceptibility (8); their use has been documented to be lower among racial/ethnic minority populations compared with non-Hispanic whites (8,9). Among African Americans, studies of diverse groups in the medical setting, including women seeking general medical services (10), cancer patients (11), and people with a family history of cancer (12), have established consistently low levels of awareness of genetic testing for early identification of cancer risk compared with non-Hispanic whites. Although studies of genetic testing specifically among Hispanics are few, analyses reveal low levels of awareness and use of genetic cancer screening (8,9,11), much of which has been shown to be related to education levels, nativity, or length of residence in the United States (8,13).

Genetic tests are increasingly being offered as direct-to-consumer (DTC) services through the Internet and other venues, providing people access to these tests without the involvement of a health care professional (14,15). For example, nutrigenomic testing, which involves testing for genes associated with common diseases, is becoming increasingly common; these results are used, together with information on diet and lifestyle habits provided by the test subject, to assess potential health risks and inform recommendations on behavioral changes, diet, or nutritional supplements (14-16). Of concern and of potential harm to consumers are 1) limited regulations and guidelines for pretest and posttest counseling, 2) inconsistencies in the accreditation of the laboratories involved, and 3) inconsistencies in medical advice regarding interpretation of results (14). In addition, a report by the US Government Accountability Office (GAO) highlighted concerns about nutrigenomic tests, stressing that such tests may mislead consumers by making vague predictions about their health risks (17). A more recent report from the GAO also highlights concerns about DTC genetic tests (18), including deceptive marketing of the capacities of such tests, lack of standardization across companies, and erroneous advice, concluding that test results are misleading and of little use to consumers. For example, given that companies test for different risk markers, different companies may provide different results regarding disease risk for the same person. In addition, because prevalence of genetic variants may differ by population or ancestry, it is not clear how different tests may benefit people from different racial/ethnic minority populations (18) or admixed populations, such as Puerto Ricans. Furthermore, the tests poorly predict disease risk and may not account for gene-gene interactions and gene-environment interactions.

Demographic differences between people who are aware of and use genetic tests have been observed for both DTC tests and for tests administered in clinical settings (13,14,19); such differences may contribute to health disparities among population subgroups. Information on public awareness, interest, or use of genetic tests is generally limited and is nonexistent for the population of Puerto Rico. This information is necessary to better understand public demand for these tests and will provide baseline data to track consumer awareness of and demand for DTC genetic tests. In addition, better knowledge is necessary to develop educational interventions, increase awareness, and improve decision making about these tests. Given the limited data on genetic testing among Hispanic populations and the lack of data for Puerto Rico, our study aimed to determine the prevalence and correlates of DTC genetic test awareness and the prevalence of genetic test use in a population-based sample of adults surveyed for the Health Information National Trends Survey (HINTS) conducted in Puerto Rico in 2009.

## Methods

### Study design and study population

We performed a secondary data analysis from a sample of adults aged 18 years or older who participated in the cross-sectional 2009 HINTS survey conducted in Puerto Rico (HINTS-PR). Data collection procedures for the 2009 HINTS-PR survey have been described in detail elsewhere (20). In brief, the 2007-2008 HINTS survey used for the mainland HINTS data collection (21) was fielded in Puerto Rico from April to June 2009, through the Puerto Rico Behavioral Risk Factor Surveillance System telephone research center. Data were collected via random-digit dialing and a computer-assisted telephone interview system; the stratified sampling frame represented 8 geographic regions designated by the Puerto Rico Department of Health. Within each stratum, sampled telephone numbers were selected with equal probability. Nonworking and nonresidential numbers were eliminated from the sample. Trained bilingual Puerto Rican interviewers collected 639 interviews from 837 households screened (weighted extended interview response rate, 76%). Of these, 611 respondents (96%) provided complete information on DTC test awareness and were included in our analysis. All of these 611 respondents also had complete information on age and sex, and although 2 were missing information on genetic test use, their data were retained for the study.

The main outcome variables included in our analysis were awareness of DTC genetic tests and use of genetic tests, assessed with the following questions: "Genetic tests that analyze your DNA, diet, and lifestyle for potential health risks are currently being marketed by companies directly to consumers. Have you heard or read about these genetic tests?" and "Have you ever had a genetic test?" Study covariates included demographic characteristics such as age, sex, education, marital status, and employment status. Information on health insurance coverage and personal and family history of cancer was also assessed. Other covariates of interest included body mass index, daily consumption of 5 or more servings of fruit or vegetables, and having a regular health care provider. Current smokers were defined as people who had smoked at least 100 cigarettes in their lifetime and continued to smoke at the time of the interview.

We also assessed information about health, cancer-specific information seeking, and sources of information. Respondents were asked, "Have you ever looked for information about health or medical topics from any source?" and "Have you ever looked for cancer information from any source?" Respondents who sought health or cancer information were asked to identify the source: "The most recent time you looked for information about health or medical topics, where did you go first?" Responses were categorized into 4 groups: Internet, printed materials (eg, brochures, pamphlets, newspapers, books), health care professionals, and other (eg, interpersonal contact, TV, radio, telephone, health organizations).

### Data analysis

All analyses were weighted to estimate standard errors of point estimates for the complex survey data by using jackknife repetition as an estimator. Data were weighted according to total estimates of sex, age, education, and marital status obtained from the American Community Survey for Puerto Rico (20) to provide representative estimates of the adult population in Puerto Rico. We calculated descriptive statistics for all variables included in this analysis. We conducted bivariate analysis to assess potential associations between demographic, health, and behavioral characteristics of respondents and awareness of genetic tests by using the Pearson χ^2^ test. On the basis of the results of the bivariate analysis, we constructed a multivariate logistic regression model to estimate odds ratios and their 95% confidence intervals for the association between covariates of interest and genetic test awareness. Interactions and goodness of fit of the model were tested with the likelihood ratio test and the Hosmer-Lemeshow test, respectively.

## Results

The majority of respondents (56%) were aware of DTC genetic tests, and approximately 4% had ever used any genetic test (Table 1). Lower awareness of DTC genetic tests was observed among men, single people, smokers, and people who had never sought health or cancer information (Table 2). Among people who were aware of genetic tests (n = 361), those who had ever looked for cancer information (n = 122) sought this information mostly via the Internet (47%), printed materials (28%), or through health care providers (16%). A similar pattern was observed for general health information seeking (data not shown).

We found no significant interactions in the multivariate model and a good model fit (Hosmer-Lemeshow test: χ^2^ = 2.50; *P* = .96). Awareness of DTC genetic tests was independently associated with marital status: people who were married or living together and those who were divorced, separated, or widowed were more likely to be aware of genetic tests than people who were never married. Current smokers were less likely than nonsmokers to be aware of genetic tests, and respondents who had ever looked for cancer information were more likely to have heard or read about DTC genetic tests than those who had not (Table 3).

## Discussion

Although our result for awareness of DTC genetic tests (56%) was higher than that reported in the United States in the 2006 HealthStyles Survey (14%) and for Hispanic (25%) and non-Hispanic (30%) respondents in the 2008 US HINTS (overall prevalence, 29%) using the same survey item (14,22,23), the lack of awareness in our sample was nevertheless high. Meanwhile, use of genetic tests in Puerto Rico (4%) was higher than that reported in the United States by the 2006 HealthStyles National Survey 1%, but lower than that reported in the 2008 US HINTS survey overall 8% and for Hispanic 9% and non-Hispanic respondents 8%. Although causal attributions cannot be established from these data, the higher awareness and use of genetic testing observed in Puerto Rico relative to mainland estimates coincides with more marketing and availability of such tests over time (24).

Similar to what has been seen in the US mainland (14), instead of health care professionals, the Internet was the primary source of information for DTC tests in our study. This source of information is of concern because a systematic analysis of websites promoting nutrigenomic services in 2006 showed that organizations that either sold or promoted these tests did not provide adequate information about nutrigenomic services and at-home genetic testing in terms of laboratory certifications, test validity or utility, or genetic counseling (16).

Even though this lack of professional information could be because limited data are available to support the products currently on the market (14), the rapid growth in the availability of health-related DTC genetic tests offers an opportunity for professional organizations to develop and promote their posture regarding the use of these tests and highlights the need for the development of consistent recommendations (15); these measures will support the tests' appropriate use as more data become available. Clinicians should advise their patients that currently available testing has little value for disease risk ascertainment but should use the opportunity to direct patient counseling toward modifiable risk factors, such as tobacco cessation (25). Government regulation regarding the use of these technologies will also be essential (26).

Consistent with the results of our study, previous research from US HINTS has identified a positive relationship between health information seeking and genetic test awareness (23). Whereas, although the 2006 HealthStyles data revealed a higher prevalence of genetic test awareness among respondents younger than 65 years and those with at least a high school education, neither age nor education were associated with awareness of genetic tests in Puerto Rico. Neither our study nor the study by Goddard and colleagues (14) identified significant associations between sex or family history of cancer and test awareness. Similar results have been observed for Hispanics in the 2000 and 2005 National Health Interview Survey, specifically for genetic testing for increased cancer risk (13,27). However, these findings are inconsistent with research reporting more awareness and use of genetic tests among women and people with a family history of cancer (5-9). For family history, the lack of association could be explained by the low proportion of people in our study with family history of cancer (low statistical power). Meanwhile, the association between smoking status and awareness of DTC genetic tests is particularly interesting given that this group is at an increased risk of multiple chronic diseases and could be a group that benefits from DTC testing in the future, if the benefit of testing is established.

Health disparities exist in cancer occurrence by race/ethnicity (28). Some of these disparities may be explained by differences in lifestyle and environmental or genetic risk factors across populations. Although we do not focus our analysis on genetic testing specifically for cancer risk, genetic cancer screening may provide needed motivation and opportunities for prevention because people with basic knowledge about inherited cancer risk and awareness of genetic testing for cancer susceptibility mutations may make more informed choices about their health care services (27). Although Puerto Ricans living in the United States show the highest level of awareness of genetic testing for cancer risk compared with other Hispanic subgroups (13,27), socioeconomic and racial/ethnic disparities in use of genetic counseling and testing for cancer susceptibility continue to exist (8,9,19). One explanation for the lower awareness and use of genetic tests for cancer risk in racial/ethnic minority populations, particularly Hispanics, is that they are less exposed to health information through the health care system partially because of language barriers (8). Low awareness about genetic testing among Hispanics may increase cancer disparities in this group (29). Thus, to guard against the possibility of tests becoming a source of health disparity (29), DTC and cancer-specific genetic tests that have proven health benefits in the future should be made equally available to all who might benefit from them.

Our study is subject to limitations. First, because HINTS-PR was a telephone-based survey, it includes data only from residents who have a working landline telephone. Consequently, our data may not be generalizable to the entire adult Puerto Rican population. Nonetheless, the weighted extended interview response rate in the 2009 HINTS-PR (76.4%) was higher than that reported for the 2007 US HINTS (60.4%) (30). Second, the prevalence estimates were based on self-reported information, which is subject to recall and social desirability biases. Third, the small number of respondents who had used genetic tests did not permit us to explore correlates of this behavior. Finally, our estimates of awareness of DTC tests and use of genetic tests may be inaccurate if respondents did not fully understand or misinterpreted the study questions. In fact, even though the research question used to determine DTC genetic test awareness included a brief explanation of these tests, some people may not understand what a genetic test is; this clarity issue should be carefully addressed in future studies.

To our knowledge, this study provides the first published data on the awareness of DTC genetic tests and use of genetic tests in Puerto Rico. These baseline data are valuable for tracking trends in awareness of DTC tests and use of genetic tests in Puerto Rico and can inform the development of policy and educational efforts regarding the appropriate use of these tests. We expect that the proportion of people who are aware of and use these tests will continue to increase, as more of these tests become available, as their marketing increases, and as the Internet and other sources of information become more widely available to the general population. Given the lack of clear benefits of DTC genetic tests to the general population, educational interventions should be developed to increase awareness and specific knowledge regarding the appropriate use of DTC genetic tests among people who are already aware of their existence (primary audience). As a clear message about these tests is in circulation, people who are unaware (secondary audience) may start to become more adequately aware of them. Future studies should try to elucidate remaining questions in this area of genetic testing, such as the prevalence of disease-specific genetic testing in clinical settings, and other psychosocial correlates of genetic testing awareness, including attitudes, beliefs, and cultural or religious norms regarding their use.

## Figures and Tables

**Table 1:** Characteristics, Awareness of Direct-to-Consumer Genetic Tests, and Use of Genetic Tests Among Puerto Rican Adults (n = 611), Health Information National Trends Survey, 2009

**Characteristic**	n	% (95% CI)^a^
**Sex**		
Female	429	52.9 (51.4-54.5)
Male	182	47.1 (45.5-48.6)
**Age, y**		
18-39	115	39.9 (36.5-43.3)
40-59	191	35.2 (31.3-39.2)
≥60	305	24.9 (23.0-26.7)
**Marital status^b^**		
Single, never married	117	31.3 (27.6-35.1)
Married/living together	306	49.0 (45.5-52.6)
Separated/widowed/divorced	170	19.9 (16.0-23.2)
**Education^c^**		
Less than high school diploma	164	23.5 (18.6-28.3)
High school/vocational diploma	167	32.0 (26.4-37.7)
College or more	259	44.5 (40.8-48.3)
**Employed^b^**		
Yes	164	40.2 (35.7-44.6)
No	429	59.8 (55.4-64.3)
**Have health insurance coverage**		
Yes	582	93.0 (90.1-95.8)
No	29	7.0 (4.2-9.9)
**Body mass index, kg/m^2^ d**		
<25.0 (underweight/normal)	212	34.8 (29.7-39.8)
25.0-29.9 (overweight)	194	31.8 (26.8-36.9)
≥30.0 (obese)	174	33.4 (27.9-38.8)
**Current smoker^e^**		
Yes	128	16.9 (12.4-21.4)
No	474	83.1 (78.6-87.6)
**Heard of direct-to-consumer genetic tests^f^**		
Yes	361	55.8 (48.4-63.2)
No	250	44.2 (36.8-51.6)
**Ever had a genetic test^g^**		
Yes	25	4.3 (1.6-7.0)
No	584	95.7 (93.0-98.4)

Abbreviation: CI, confidence interval.

a Percentages are weighted.

b 18 missing values.

c 21 missing values.

d 31 missing values.

e 9 missing values.

f Standard error = 3.68 using jackknife repetition as an estimator.

g 2 missing values. Standard error = 1.36 using jackknife repetition as an estimator.

**Table 2:** Prevalence of Direct-to-Consumer Genetic Test Awareness, by Selected Characteristics, Among Puerto Rican Adults (n = 611), Health Information National Trends Survey, 2009

Characteristic	Test Awareness		*P* Value^b^

	n^a^	% (95% CI)	
**Sex**			
Female	266	63.3 (55.8-70.7)	.01
Male	95	47.3 (35.7-58.9)	
**Age, y**			
18-39	69	57.1 (46.0-68.3)	.83
40-59	117	53.7 (42.8-64.7)	
≥60	175	56.5 (50.3-62.6)	
**Marital status**			
Single, never married	63	47.1 (35.3-59.0)	.03
Married/living together	188	56.9 (48.4-65.4)	
Separated/widowed/divorced	100	66.6 (56.4-76.9)	
**Education**			
<High school	84	53.4 (43.4-63.4)	.16
High school/vocational diploma	91	46.8 (33.5-60.0)	
College or more	174	63.2 (52.0-74.3)	
**Employed**			
Yes	103	54.7 (42.1-67.3)	.79
No	248	56.5 (48.6-64.3)	
**Have health insurance coverage**			
Yes	345	55.8 (48.1-63.5)	.98
No	16	55.5 (25.6-85.3)	
**Personal history of cancer**			
Yes	28	66.0 (47.2-84.8)	.25
No	322	55.2 (47.4-63.0)	
**Family history of cancer**			
Yes	228	57.8 (49.5-66.1)	.33
No	122	51.8 (40.1-63.5)	
**Body mass index, kg/m^2^**			
<25.0 (underweight/normal)	130	61.2 (50.8-71.6)	.40
25.0-29.9 (overweight)	119	55.5 (43.8-67.2)	
≥30.0 (obese)	96	50.4 (37.9-62.9)	
**Current smoker**			
Yes	72	42.9 (33.0-52.8)	.02
No	282	58.0 (49.6-66.4)	
**Have regular health care provider**			
Yes	292	58.3 (50.6-66.0)	.20
No	69	48.5 (34.4-62.6)	
**≥5 Servings fruits or vegetables daily**			
Yes	70	60.3 (44.8-62.6)	.49
No	291	54.9 (47.2-62.6)	
**Ever looked for information on health topics**			
Yes	150	67.0 (57.9-76.2)	.003
No	211	50.2 (41.3-59.1)	
**Ever looked for cancer information**			
Yes	122	72.0 (61.2-82.7)	.001
No	239	50.2 (40.8-58.7)	

Abbreviation: CI, confidence interval.

a Not all categories add to total for awareness (n = 361) because of missing values.

b Calculated by using the Pearson χ^2^ test.

**Table 3:** Odds of Genetic Testing Awareness, by Demographic Characteristics, Among Puerto Rican Adults, Health Information National Trends Survey, 2009

**Characteristic**	Odds Ratio (95% CI)
**Sex**	
Male	1 [Reference]
Female	1.41 (0.79-2.50)
**Age, y**	
18-39	1 [Reference]
40-59	0.81 (0.46-1.43)
≥60	0.99 (0.54-1.83)
**Marital status**	
Single, never married	1 [Reference]
Separated/widowed/divorced	2.37 (1.07-5.24)
Married/living together	1.76 (1.02-3.03)
**Ever looked for information on health topics**	
No	1 [Reference]
Yes	1.46 (0.90-2.38)
**Ever looked for cancer information**	
No	1 [Reference]
Yes	2.04 (1.06-3.93)
**Current smoking**	
No	1 [Reference]
Yes	0.52 (0.32-0.84)

Abbreviation: CI, confidence interval.

## References

[1] Butler D (2010). Human genome at ten: science after the sequence. Nature.

[2] Burke W (2002). Genetic testing. N Engl J Med.

[3] Chung WK (2007). Implementation of genetics to personalize medicine. Gend Med.

[4] Robson ME, Storm CD, Weitzel J, Wollins DS, Offit K, American Society of Clinical Oncology (2010). American Society of Clinical Oncology policy statement update: genetic and genomic testing for cancer susceptibility. J Clin Oncol.

[5] Durfy SJ, Bowen DJ, McTiernan A, Sporleder J, Burke W (1999). Attitudes and interest in genetic testing for breast and ovarian cancer susceptibility in diverse groups of women in western Washington. Cancer Epidemiol Biomarkers Prev.

[6] Kinney AY, Choi YA, DeVellis B, Kobetz E, Millikan RC, Sandler RS (2000). Interest in genetic testing among first-degree relatives of colorectal cancer patients. Am J Prev Med.

[7] Bottorff JL, Ratner PA, Balneaves LG, Richardson CG, McCullum M, Hack T (2002). Women's interest in genetic testing for breast cancer risk: the influence of sociodemographics and knowledge. Cancer Epidemiol Biomarkers Prev.

[8] Pagán JA, Su D, Li L, Armstrong K, Asch DA (2009). Racial and ethnic disparities in awareness of genetic testing for cancer risk. Am J Prev Med.

[9] Wideroff L, Vadaparampil ST, Breen N, Croyle RT, Freedman AN (2003). Awareness of genetic testing for increased cancer risk in the year 2000 National Health Interview Survey. Community Genet.

[10] Donovan KA, Tucker DC (2000). Knowledge about genetic risk for breast cancer and perceptions of genetic testing in a sociodemographically diverse sample. J Behav Med.

[11] Kinney AY, Croyle RT, Dudley WN, Baley CA, Pelias MK, Neuhausen SL (2001). Knowledge, attitudes, and interest in breast-ovarian cancer gene testing: a survey of a large African-American kindred with a BRCA1 mutation. Prev Med.

[12] Lacour RA, Daniels MS, Westin SN, Meyer LA, Burke CC, Burns KA (2008). What women with ovarian cancer think and know about genetic testing. Gynecol Oncol.

[13] Heck JE, Franco R, Jurkowski JM, Sheinfeld Gorin S (2008). Awareness of genetic testing for cancer among United States Hispanics: the role of acculturation. Community Genet.

[14] Goddard KA, Moore C, Ottman D, Szegda KL, Bradley L, Khoury MJ (2007). Awareness and use of direct-to-consumer nutrigenomic tests, United States, 2006. Genet Med.

[15] Goddard KA, Robitaille J, Dowling NF, Parrado AR, Fishman J, Bradley LA (2009). Health-related direct-to-consumer genetic tests: a public health assessment and analysis of practices related to Internet-based tests for risk of thrombosis. Public Health Genomics.

[16] Sterling R (2008). The on-line promotion and sale of nutrigenomic services. Genet Med.

[17] (2006). Testimony before the Special Committee on Aging, US Senate. Nutrigenetic testing: tests purchased from four web sites mislead customers.

[18] (2010). Testimony before the Subcommittee on Oversight and Investigations, Committee on Energy and Commerce, House of Representatives. Direct-to-consumer genetic tests: misleading test results are further complicated by deceptive marketing and other questionable practices.

[19] Suther S, Kiros GE (2009). Barriers to the use of genetic testing: a study of racial and ethnic disparities. Genet Med.

[20] Tortolero-Luna G, Finney Rutten LJ, Hesse BW, Davis T, Kornfeld J, Sanchez M (2010). Health and cancer information seeking practices and preferences in Puerto Rico: creating an evidence base for cancer communication efforts. J Health Commun.

[21] Survey instruments. Health Information National Trends Survey.

[22] Have you heard or read about any genetic tests? 2007 data. Health Information National Trends Survey.

[23] Lee JA, Lustria MLA Linking health information seeking, genetic testing and public awareness of the Genetic Information Nondiscrimination Act (GINA) in the US. Poster presented at: HINTS Data Users Conference.

[24] (2004). Workshop summary. Direct to consumer (DTC) advertising of genetic tests.

[25] Manolio TA (2010). Genomewide association studies and assessment of the risk of disease. N Engl J Med.

[26] Annes JP, Giovanni MA, Murray MF (2010). Risks of presymptomatic direct-to-consumer genetic testing. N Engl J Med.

[27] Vadaparampil ST, Wideroff L, Breen N, Trapido E (2006). The impact of acculturation on awareness of genetic testing for increased cancer risk among Hispanics in the year 2000 National Health Interview Survey. Cancer Epidemiol Biomarkers Prev.

[28] Howe HL, Wu X, Ries LA, Cokkinides V, Ahmed F, Jemal A (2006). Annual report to the nation on the status of cancer, 1975-2003, featuring cancer among US Hispanic/Latino populations. Cancer.

[29] Rotimi CN, Jorde LB (2010). Ancestry and disease in the age of genomic medicine. N Engl J Med.

[30] Health Information National Trends Survey 2007: final report.

